# Supplementary material for: ProteinShader: illustrative rendering of macromolecules
Source: BMC Struct Biol. 2009 Mar 30;9:19. doi: 10.1186/1472-6807-9-19 (PMC2672931; doi:10.1186/1472-6807-9-19)
Supplement: Additional file 1 — ProteinShader program without source code. This compressed file contains the complete ProteinShader program including associated libraries, but no source code. A README.txt file gives an overview of the ProteinShader distribution, and the index.html file in the help subdirectory has directions on getting started with the program as well as a set of tutorials. [file 1472-6807-9-19-S1.zip › ProteinShader-beta-0_9_4-binary/help/api/org/proteinshader/graphics/typography/GLF2Header.html]

GLF2Header (ProteinShader API)


|  |  |  |
| --- | --- | --- |
| **Overview** \| **Package** \| **Class** \| **Use** \| **Tree** \| **Deprecated** \| **Index** \| **Help** | |  |
| **PREV CLASS**   **NEXT CLASS** | **FRAMES**    **NO FRAMES**     **All Classes** |  |
| SUMMARY: NESTED \| FIELD \| CONSTR \| METHOD | DETAIL: FIELD \| CONSTR \| METHOD |  |


---


## org.proteinshader.graphics.typography Class GLF2Header

```
java.lang.Object
  org.proteinshader.graphics.typography.GLF2Header
```

---

``` public class GLF2Header extends Object ```

Stores information from the header of a '.glf' file. See comments in
class GLF2GlyphSetFactory for an explanation of what a '.glf' file is.

---

| **Field Summary** | |
| --- | --- |
| `static int` | `BYTES_PER_PIXEL`             The mipmap uses two bytes per pixel (luminescence and alpha). |
| `static int` | `CHAR_INFO_BYTES`             Each character info block in a '.glf' file has 24 bytes. |
| `static int` | `END_INDEX`             The end char integer starts at index 16 of the header. |
| `static int` | `HEADER_BYTES`             The header in a '.glf' file has 24 bytes. |
| `static int` | `HEIGHT_INDEX`             The bitmap height integer starts at index 8 of the header. |
| `static int` | `START_INDEX`             The start char integer starts at index 12 of the header. |
| `static int` | `WIDTH_INDEX`             The bitmap width integer starts at index 4 of the header. |
| `static int` | `X1_INDEX`             The bitmap width integer starts at index 8 of the header. |
| `static int` | `X2_INDEX`             The start char integer starts at index 16 of the header. |
| `static int` | `Y1_INDEX`             The bitmap height integer starts at index 12 of the header. |
| `static int` | `Y2_INDEX`             The end char integer starts at index 20 of the header. |


**Constructor Summary**

`GLF2Header(String filename,
int bitmapWidth,
int bitmapHeight,
int startChar,
int endChar)`
  
          Constructs a GLF2Header.


| **Method Summary** | |
| --- | --- |
| `int` | `getBitmapHeight()`             Returns the height of the bitmap. |
| `int` | `getBitmapWidth()`             Returns the width of the bitmap. |
| `int` | `getEndChar()`             Returns the end char (the last character in the glyph set). |
| `String` | `getFilename()`             Returns the name of the '.glf' file the header is taken from. |
| `int` | `getStartChar()`             Returns the start char (the first character in the glyph set). |

**Methods inherited from class java.lang.Object**

`clone, equals, finalize, getClass, hashCode, notify, notifyAll, toString, wait, wait, wait`

**Field Detail**

### HEADER\_BYTES

```
public static final int HEADER_BYTES
```

:   The header in a '.glf' file has 24 bytes.

    **See Also:**: Constant Field Values

---


### WIDTH\_INDEX

```
public static final int WIDTH_INDEX
```

:   The bitmap width integer starts at index 4 of the header.

    **See Also:**: Constant Field Values

---


### HEIGHT\_INDEX

```
public static final int HEIGHT_INDEX
```

:   The bitmap height integer starts at index 8 of the header.

    **See Also:**: Constant Field Values

---


### START\_INDEX

```
public static final int START_INDEX
```

:   The start char integer starts at index 12 of the header.

    **See Also:**: Constant Field Values

---


### END\_INDEX

```
public static final int END_INDEX
```

:   The end char integer starts at index 16 of the header.

    **See Also:**: Constant Field Values

---


### CHAR\_INFO\_BYTES

```
public static final int CHAR_INFO_BYTES
```

:   Each character info block in a '.glf' file has 24 bytes.

    **See Also:**: Constant Field Values

---


### X1\_INDEX

```
public static final int X1_INDEX
```

:   The bitmap width integer starts at index 8 of the header.

    **See Also:**: Constant Field Values

---


### Y1\_INDEX

```
public static final int Y1_INDEX
```

:   The bitmap height integer starts at index 12 of the header.

    **See Also:**: Constant Field Values

---


### X2\_INDEX

```
public static final int X2_INDEX
```

:   The start char integer starts at index 16 of the header.

    **See Also:**: Constant Field Values

---


### Y2\_INDEX

```
public static final int Y2_INDEX
```

:   The end char integer starts at index 20 of the header.

    **See Also:**: Constant Field Values

---


### BYTES\_PER\_PIXEL

```
public static final int BYTES_PER_PIXEL
```

:   The mipmap uses two bytes per
    pixel (luminescence and alpha).

    **See Also:**: Constant Field Values


**Constructor Detail**

### GLF2Header

```
public GLF2Header(String filename,
                  int bitmapWidth,
                  int bitmapHeight,
                  int startChar,
                  int endChar)
```

:   Constructs a GLF2Header.

    **Parameters:**: `filename` - the name of the '.glf' file.: `bitmapWidth` - the width of the bitmap in the '.glf' file.: `bitmapHeight` - the height of the bitmap in the '.glf' file.: `startChar` - the first character in the '.glf' file.: `endChar` - the last character in the '.glf' file.


**Method Detail**

### getFilename

```
public String getFilename()
```

:   Returns the name of the '.glf' file the header is taken from.

    :   **Returns:**: The name of the '.glf'.

---


### getBitmapWidth

```
public int getBitmapWidth()
```

:   Returns the width of the bitmap.

    :   **Returns:**: The width in pixels.

---


### getBitmapHeight

```
public int getBitmapHeight()
```

:   Returns the height of the bitmap.

    :   **Returns:**: The height in pixels.

---


### getStartChar

```
public int getStartChar()
```

:   Returns the start char (the first character in the glyph set).

    :   **Returns:**: The start char as an integer.

---


### getEndChar

```
public int getEndChar()
```

:   Returns the end char (the last character in the glyph set).

    :   **Returns:**: The end char as an integer.


---


|  |  |  |
| --- | --- | --- |
| **Overview** \| **Package** \| **Class** \| **Use** \| **Tree** \| **Deprecated** \| **Index** \| **Help** | |  |
| **PREV CLASS**   **NEXT CLASS** | **FRAMES**    **NO FRAMES**     **All Classes** |  |
| SUMMARY: NESTED \| FIELD \| CONSTR \| METHOD | DETAIL: FIELD \| CONSTR \| METHOD |  |


---

# *Copyright © 2007-2008*
